# Transient receptor potential melastatin 2 regulates neutrophil extracellular traps formation and delays resolution of neutrophil-driven sterile inflammation

**DOI:** 10.1186/s12950-023-00334-1

**Published:** 2023-02-21

**Authors:** Xue Cao, Yanhong Li, Yubin Luo, Tianshu Chu, Hang Yang, Ji Wen, Yi Liu, Yi Zhao, Martin Herrmann

**Affiliations:** 1 Department of Rheumatology and Immunology, West China Hospital, Sichuan University, Chengdu, Sichuan China; 2 Department of Rheumatology and Immunology, Henan Provincial People’s Hospital, People’s Hospital of Zhengzhou University, People’s Hospital of Henan University, Zhengzhou, Henan China; 3 Department of Internal Medicine 3, Rheumatology and Immunology, Friedrich-Alexander- University Erlangen-Nürnberg (FAU), Erlangen, Germany; 4 Department of Rheumatology & Immunology, West China Hospital, Sichuan University, No.37, Guoxue Alley, Chengdu, 610041 Sichuan China

**Keywords:** TRPM2, Neutrophil extracellular traps, Sterile inflammation, MSU crystals, ROS, NET formation

## Abstract

The formation of neutrophil extracellular traps (NETs) is a process releasing into the extracellular space networks of chromatin fibers decorated with granular proteins. It is implicated in infection-related as well as sterile inflammation. Monosodium urate (MSU) crystals serve as damage-associated molecular pattern (DAMP) in various conditions of disease. Formation of NETs or aggregated NETs (aggNETs) orchestrates initiation and resolution of MSU crystals-triggered inflammation, respectively. Elevated intracellular calcium levels and the generation of reactive oxygen species (ROS) are crucial for the formation of MSU crystal-induced NETs. However, the exact signaling pathways involved are still elusive. Herein, we demonstrate that the ROS-sensing, non-selective calcium-permeable channel transient receptor potential cation channel subfamily M member 2 (TRPM2) is required for a full-blown MSU crystal-induced NET formation. Primary neutrophils from TRPM2^−/−^ mice showed reduced calcium influx and ROS production and, consequently a reduced formation of MSU crystal-induced NETs and aggNETs. Furthermore, in TRPM2^−/−^ mice the infiltration of inflammatory cells into infected tissues and their production of inflammatory mediators was suppressed. Taken together these results describe an inflammatory role of TRPM2 for neutrophil-driven inflammation and identify TRPM2 as potential target for therapeutic intervention.

## Summary sentence

Pathophysiological role of TRPM2 in MSU crystals-induced inflammation by affecting NET formation.

## Introduction

Neutrophils are the most abundant leukocytes in the human circulation and perform a protective role in infectious diseases by constituting the first line of innate immune to combat microbial infection [1]. The antimicrobial functions of neutrophils are mainly mediated by phagocytosis, degranulation, generation of reactive oxygen species (ROS) and the formation of NETs [2, 3]. The NETs are composed of modified, decondensed chromatin decorated with cytoplasmic and granular proteins like neutrophil elastase (NE), myeloperoxidase (MPO), and other antibacterial proteins. Immobilizing, neutralizing, and killing pathogens NETs play a protective role in the immune defense [4, 5], however, they are also implicated in non-infectious sterile inflammations [6]. Over the years, it has become increasingly clear that only a specific subpopulation of neutrophils can make NETs, especially during the sterile inflammation [7]. Sterile inflammation induced by DAMPs is a basis for the etiopathogenesis of several autoimmune diseases [8]. MSU crystals robustly induce ROS- and Ca^2+^-dependent NET formation that is accompanied by sterile inflammation. Exposure to MSU crystals activates neutrophils that respond with a rapid formation of NETs precipitating acute inflammatory reactions. Interestingly, in the presence of high densities of neutrophils, NETs tend to aggregate and form aggNETs that proteolytically degrade cytokines and chemokines by the action of various serine proteases and thus initiate the resolution of inflammation [9, 10]. Identification of a target that regulates MSU crystal-induced NET formation is of far-reaching significance for conditions of neutrophil-driven inflammation.

TRPM2 is a nonselective Ca^2+^-permeable membrane cation channel that converts ROS- induced oxidative stress into Ca^2+^ signals [11]. It is highly expressed on the surfaces of neutrophils, macrophages, monocytes, lymphocytes and dendritic cells to connect with such events as inflammation, regulation of endothelial permeability, development of cancer and degenerative diseases, or induction of cell death, including apoptosis and autophagy [12–14]. Obviously, TRPM2 participates in the regulation of physiologic and pathologic immune responses in tissue sites under oxidative stress [15, 16]. Reportedly, TRPM2 regulated neutrophil migration and chemotaxis [17, 18].

However, the role of TRPM2 during the formation NETs is still elusive. The present study focused on the role of TRPM2 in MSU crystals-induced NET formation and inflammation. In this study, we used primary neutrophils and a murine model of MSU crystals-induced inflammation to analyze the pathophysiological role of TRPM2 in MSU crystals-induced NET formation in vitro and in vivo.

## Materials and methods

### Mice

All in vitro and in vivo experiments were performed using 6- to 8-week-old wild type (WT) or TRPM2^−/−^ mice on the C57BL/6 background. Male WT mice were purchased from Huashuo animal center (Chengdu, China). TRPM2^−/−^ mice were obtained from State Key Laboratory of Biotherapy at Sichuan University and maintained at 25 °C with 12 h light and dark cycles. All animal experiments were performed under the protocol approved by Animal Ethics Committee in China.

### MSU crystals

MSU crystals were produced as described previously [10, 19]. We dissolved 10 mM uric acid (Merck KGaA) and 154 mM NaCl (Merck KGaA) to pH 7.2 and stirred it for 3d. We dried the resulting crystals under sterile conditions at 180 °C for 2 h after ethanol washing, and stored them in PBS (pH 7.0).

### NET formation in vitro

For in vitro experiments, bone marrow-derived neutrophils from of WT or TRPM2^−/−^ mice were cultured at 5 × 10 ^6^ cells/ml with 200 μg/ml MSU crystals for 2 h at 37 °C. Samples were fixed with 4% paraformaldehyde and NETs were stained with DAPI (Invitrogen, California, USA) and antibodies to NE (Bioss, Beijing, China), MPO (R&D, Minnesota, USA) and analyzed by immune fluorescence microscopy (ZEISS, Oberkochen, Germany). We collected photos in each field of view and calculated the percent of DNA in NETs area by Adobe Photoshop CC 2018.

### NET formation in animal experiments

WT or TRPM2^−/−^ mice were anaesthetized with chloral hydrate and challenged with subcutaneous injection of 3 ml sterile air into the back to form air pouches. Three days later, another 2 ml of sterile air was injected into the preformed air pouch. After another 24 h, 5 mg MSU crystals in PBS were injected into the air pouches. The lavage fluid was collected by flushing the pouch with 2 ml sterile PBS 24 h after administration of the MSU crystals. The size of the MSU crystal aggregates formed in the air pouches is referred to as “Tophus score”.

After the surgical removal of the air bags the specimens were scored blinded by checking size and number of the amorphous material that had clumped together in pale white lumps. Twenty milligrams of MSU crystals in 1 ml PBS were intraperitoneal injected into WT or TRPM2^−/−^ mice pretreated with thioglycolate (Sigma, Missouri, USA). After 24 h, the aggregation of MSU crystals in the abdominal cavity was monitored after surgical dissection of the peritoneum. 0.6 mg of MSU crystals were administered in 30 μl PBS into the foot pads of WT or TRPM2^−/−^ mice. As control, the contralateral foot pads were injected with 30 μl sterile PBS. Paw swelling was measured with an electronic vernier scale by a blinded experimenter at the time points indicated.

### Measurement of extracellular DNA

Extracellular DNA was quantified by using Quant-iT® PicoGreen® dsDNA Reagent (Invitrogen, California, USA). Briefly, PicoGreen was added to the supernatant of isolated neutrophils incubated with 200 μg/ml MSU crystals or air pouch lavage fluid in a black 98- well microplate. The fluorescence was quantified employing a fluorescence microplate reader with 485/535 nm excitation/emission. The concentrations of extracellular DNA were calculated based on a standard curve provided in kit (0-1000 ng/mL).

### Measurement of calcium influx and ROS production

Mouse bone marrow neutrophils were incubated with 2.5 μM Fura-2 AM (Beyotime, Shanghai, China) or 10 μM DCFH-DA (Sigma, Missouri, USA) for 30 min at 37 °C, followed by stimulation with 200 μg/ml MSU crystals. The fluorescence intensities of Fura-2 or DCF were recorded employing a fluorescence microplate reader with 340/380 nm or 485/535 nm excitation/emission.

### Flow cytometry and ELISA analysis

The air pouch lavage fluids were harvested as described above and centrifuged at 1800 rpm for 5 min. The pellets were resuspended with 3% BSA in PBS and stained with PerCP-Cy5.5- conjugated anti-Ly6G(BD, New Jersey, USA) for 20 min at 4 °C and then analyzed by flow cytometry (BD, New Jersey, USA).

The air pouch lavage fluid was collected for cytokines measurement. IL-1β, IL-6 and TNF- α levels were detected by ELISA (Bio-Swamp, Nantong, China) according to the manufacturers’ instructions.

### Histology, immunohistochemistry and μCT scanning

Using standard protocols paraffin sections of the air pouches and foot pads were prepared for analyses by histology. They were stained with hematoxylin/eosin (H&E). For immunohistochemistry, the tissue sections were stained with anti-MPO antibodies (R&D, Minnesota, USA). Hematoxylin served as counterstain.

The paws were assessed using μCT (PerkinElmer, Massachusetts, USA). The acquisition parameters were: X Ray voltage = 90 kV, intensity = 88 μA, field of view (FOV) = 25 mm, pixel size = 50 μm and at least 1500 layers. Three-dimensional reconstruction of the paws was performed employing the “Analyze 12.0” μCT analysis software.

### Statistical analyses

All data were presented as Mean ± SD or Mean ± SEM as indicated. Two-tailed Student’s t tests were used for comparisons of independent experiments. The statistical significances among multiple groups were determined by one-way ANOVA. All statistical analyses were performed using GraphPad Prism®. According to GP formatting *p* < 0.05, *p* < 0.01, and *p* < 0.001 were considered significant, very significant and extremely significant, respectively.

## Results

### TRPM2^−/−^ neutrophils show reduced MSU crystals-induced NET formation

**Fig. 1:**
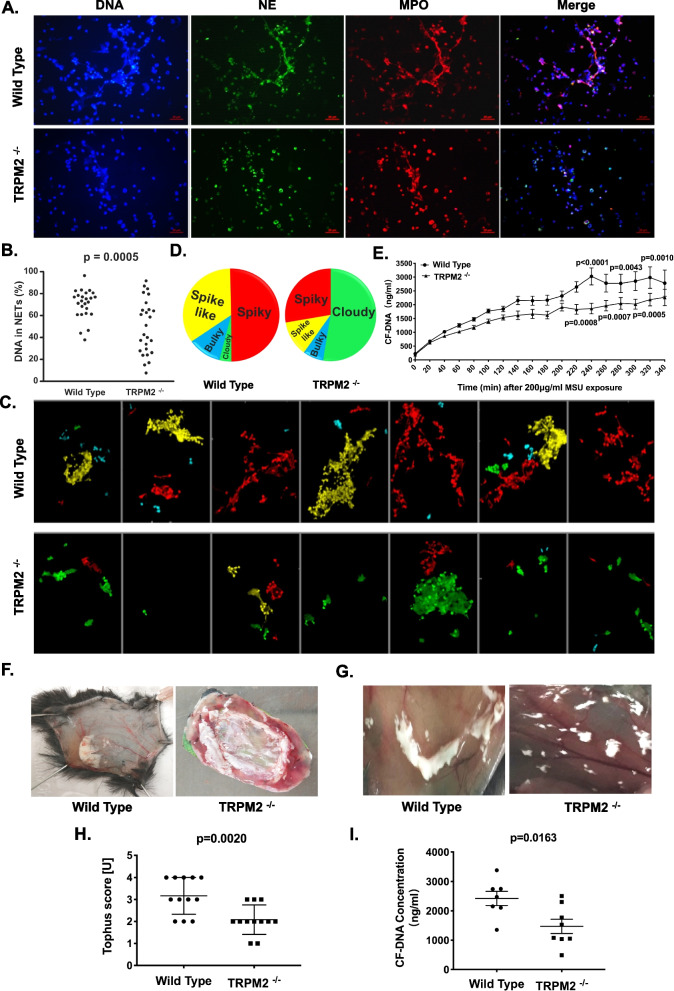
TRPM2 is involved in MSU crystals-induced NET formation and aggregation. **A** Representative fluorescence microscopy images of neutrophils of WT or TRPM2^−/−^ mice that had been treated with MSU crystals for 2 h. NETs were fixed and stained with DNA (DAPI), MPO and NE. **B** The scatter plots shows quantification of NETs in WT and TRPM2^−/−^ neutrophils. Scale bars, 20 μm. *p* = 0.0005. **C** Representative NETs morphologyies, such as spiky (red), cloudy (green), spike-like (yellow) and bulky (blue) artificially stained from a blinded examiner. **D** The pie charts describe the classification of the NETs morphology. **E** The dynamic changes of the levels of extracellular DNA in the supernatants of neutrophils derived from WT and TRPM2^−/−^ mice for 340 min (20 min intervals) after addition of MSU crystals. Mean ± SEM is displayed. The *p*-values at 220 min, 240 min, 260 min, 280 min, 300 min and 320 min are 0.0008, <0.0001, 0.0007, 0.0043, 0.0005 and 0.0010, respectively. **F** Representative photographs of aggNETs formed after injection of 5 mg MSU crystals into preformed air pouches in WT and TRPM2^−/−^ mice. **G** Representative photographs of aggNETs formed after intraperitoneal (i.p.) injection of 20 mg MSU crystals into WT and TRPM2^−/−^ mice that had been pretreated with thioglycolate 24 h before. **H** The scatter plots show the degree of NETs aggregation. Mean ± SD is displayed. *p* = 0.0020. **I** Levels of extracellular DNA in the supernatants of air pouches lavage fluid of WT and TRPM2^−/−^ mice. Mean ± SEM is displayed; *p* = 0.0163

To examine the impact of TRPM2 on NETs formation in vitro, we incubated neutrophils from WT and TRPM2^−/−^ mice with MSU crystals and analyzed them for NET formation. As shown in Fig. 1A, B, the large stretches of extracellular DNA colocalizing with NE and MPO were abundant in WT mice, but were dramatically reduced in TRPM2^−/−^ mice and exhibited a predominantly nuclear appearance. After classifying NETs into spiky which refers to a distinct fibrous network structure, spike-like which refers to a less pronounced fibrous reticular structure, bulky and cloudy NETs, we observed that neutrophils from WT and TRPM2^−/−^ mice mostly formed spiky and cloudy NETs, respectively (Fig. 1C, D). Using the Quant-iT PicoGreen assay, we quantified the extracellular DNA in the culture supernatants of neutrophils derived from WT and TRPM2^−/−^ mice. Exposure to MSU crystals caused a gradual increase of the extracellular DNA levels. In TRPM2^−/−^ mice lower extracellular DNA levels were to be detected, especially 220 to 320 min after exposure to the MSU crystals (Fig. 1E). Thus, NET formation was accompanied by an increase of soluble extracellular DNA in the culture supernatants.

The observation that the in vitro NET formation elicited by MSU crystals was reduced in neutrophils form TRPM2^−/−^ mice prompted us to explore whether such difference also occur in vivo. To this end we injected MSU crystals (I) intraperitoneally (i.p.) into mice that had been pretreated with thioglycolate or (II) into air pouches of WT and TRPM2^−/−^ mice. In the air bags and the peritonea of WT mice, aggregated amorphous materials clumped together to form large, bright white lumps containing a high number of MSU crystals. In contrast, the MSU crystals spread in TRPM2^−/−^ mice and showed a decreased tendency for aggregation (Fig. 1F, G). The difference in the tophus score between the WT and TRPM2^−/−^ mice is displayed in Fig. 1H. The extracellular DNA in the air pouches lavage fluids were significantly decreased in TRPM2^−/−^ mice (Fig. 1I). Taken together, these in vivo data were in accordance with our results in vitro. NET formation and aggregation of TRPM2^−/−^ neutrophils after stimulation with MSU crystals was reduced and the NETs showed a more cloudy appearance.

### TRPM2 mediates MSU crystals-induced calcium influx and ROS production of neutrophils

**Fig. 2:**
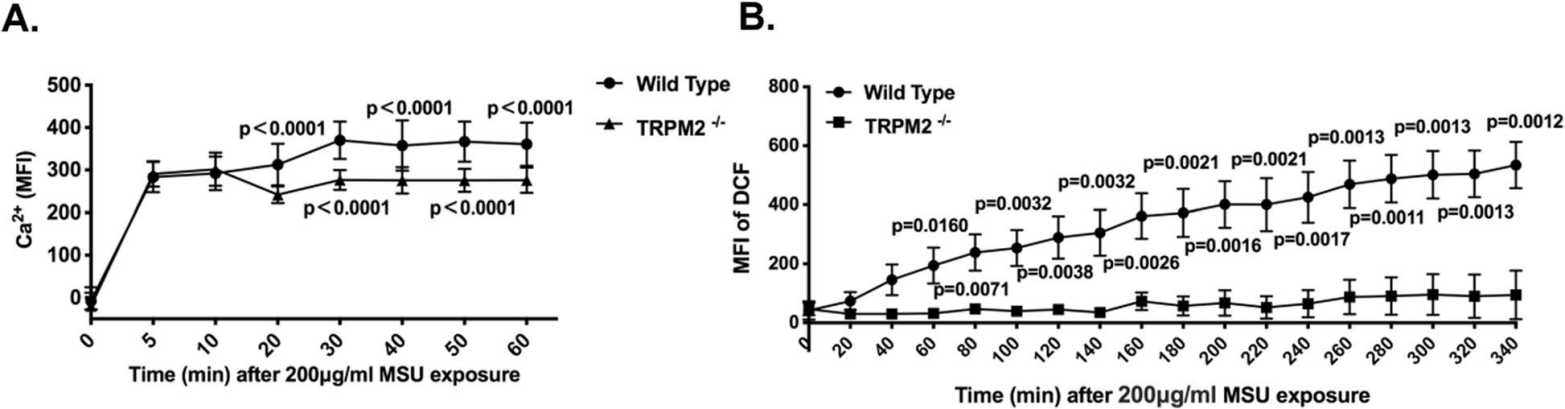
Deletion of TRPM2 reduces calcium influx and ROS production in MSU crystals-treated neutrophils. **A** Representative display of the dynamic changes of [Ca^2+^]i in WT and TRPM2^−/−^ neutrophils within 60 min after exposure to MSU crystals. Mean ± SD is displayed. The *p* values at 20-60 min are <0.0001. **B** Representative display of the ROS production in WT and TRPM2^−/−^ mice neutrophils within 340 min after exposure to MSU crystals. Mean ± SEM is displayed. The *p* values at 60 min to 340 min are 0.0160, 0.0071, 0.0032, 0.0038, 0.0032, 0.0026, 0.0021, 0.0016, 0.0021, 0.0017, 0.0013, 0.0011, 0.0013, 0.0012 and 0.0013, respectively

Calcium is established to play an important role in the initiation of NET formation [20]. To address the function of TRPM2 in the MSU crystal-induced calcium influx into neutrophils, we assessed the relative concentration of intracellular calcium ([Ca^2+^]i) using the fluorescent dye, Fura-2. Bone marrow-derived neutrophils from of WT and TRPM2^−/−^ mice were incubated with 2.5 μM Fura-2 AM at 37 °C. After the addition of the MSU crystals, the Fura-2 fluorescence was quantified by a fluorescence reader. As shown in Fig. 2A, MSU crystals- evoked increases of [Ca^2+^]i were observed within 5 min. Twenty minutes after the stimulus TRPM2^−/−^ neutrophils attenuated the [Ca^2+^]i, compared to WT neutrophils. This suggests that TRPM2 plays a key role in MSU crystals-induced Ca^2+^ influx.

It has previously been shown that MSU crystals-induced NET formation depends on the formation of ROS [21]. That’s why we employed DCF fluorescence to analyze the effect of TRPM2 on the production of ROS in neutrophils that had been treated with MSU crystals. After pre-incubation with 10 μM of the ROS sensor DCFH-DA and exposure to MSU crystals, the DCF fluorescence in neutrophils was quantified by a plate fluorescence reader. In WT neutrophils the exposure to MSU crystals caused gradually increased ROS production up to 340 min; TRPM2^−/−^ neutrophils showed a significant lower production of ROS (Fig. 2B).

These data show that TRPM2 augmented the ROS production induced in neutrophils that had been treated with MSU crystals. Hence, we suppose that the defective in MSU crystals-induced NET formation of TRPM2^−/−^ neutrophils may be due to both reduced calcium influx and ROS production.

### TRPM2 mediates infiltration and activation of inflammatory cells in MSU crystals- induced inflammation of murine air pouches

**Fig. 3:**
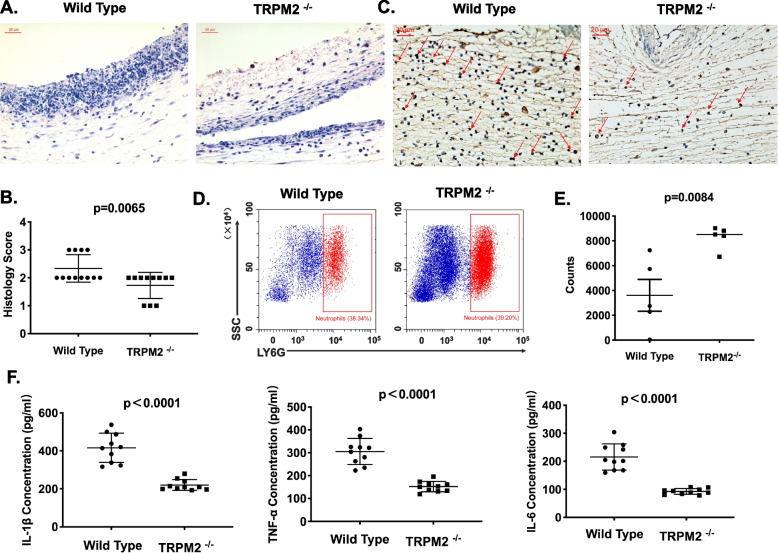
TRPM2 mediates the MSU crystal-induced inflammation in murine air pouches. **A** Representative Hematoxylin and Eosin (H&E) staining of the air pouche membranes from of WT and TRPM2^−/−^ mice treated with MSU crystals. **B** The scatter plots show the inflammatory cell infiltration into the air pouche membranes of WT and TRPM2^−/−^ mice. Scale bars, 20 μm. Mean ± SD is displayed. *p* = 0.0065. **C** Representative staining by immunohistochemistry of MPO expression in air pouche membranes. Nuclear counterstain was performed with hematoxylin. MPO-expressing cells are brown in color. Scale bars, 20 μm. **D** Representative flow cytometry plots and **E** quantification of neutrophils (Ly6G^+^) in air pouches fluids of WT and TRPM2^−/−^ mice. Mean ± SEM is displayed. *p* = 0.0084. **F** Reduced concentrations of IL-1β, IL-6 and TNF-α in air pouches fluids from TRPM2^−/−^ mice. Mean ± SD is displayed. *p* < 0.0001

Next we examined the effect of TRPM2-mediated NET formation in MSU crystals- induced neutrophilic inflammation. We injected 5 mg MSU crystals into preformed air pouches of WT and TRPM2^−/−^ mice. Twenty-four hours later we collected air pouch fluids and membranes for the analyses of inflammatory mediators and histopathology, respectively. Exposure to MSU crystals caused abundant inflammatory cells infiltration into the air pouch membranes (Fig. 3A). TRPM2^−/−^ mice exhibited much lower histopathological scores when compared to WT mice (Fig. 3B). Inflamed tissues were densely infiltrated by neutrophils. MPO, a biomarker for neutrophils in immunohistochemistry, showed a lower expression in TRPM2^−/−^ mice when compared with wild type mice (Fig. 3C). To assess if the alleviated inflammation in TRPM2^−/−^ mice was associated with neutrophils, we stained cells with PerCP- Cy5.5-conjugated anti-Ly6G in the air pouch lavage fluid and detected by flow cytometry. As shown in Fig. 3D-E, the challenge with MSU crystals caused a significantly decreased in the numbers of neutrophils (Ly6G^+^) in the lavage fluids of WT mice on account of more neutrophils forming NETs. Due to NET formation accompanied by cytokines release, the knockout of TRPM2 led to marked decreases of the prototypical inflammatory mediators IL-1β, IL-6 and TNF-α (Fig. 3F). Taken together, these data indicate that TRPM2 augments the inflammatory cascade and that targeting of TRPM2 may have a therapeutic role for neutrophil-driven conditions of inflammation.

### TRPM2^−/−^ ameliorates the inflammation in MSU crystals-induced paw edema

**Fig. 4:**
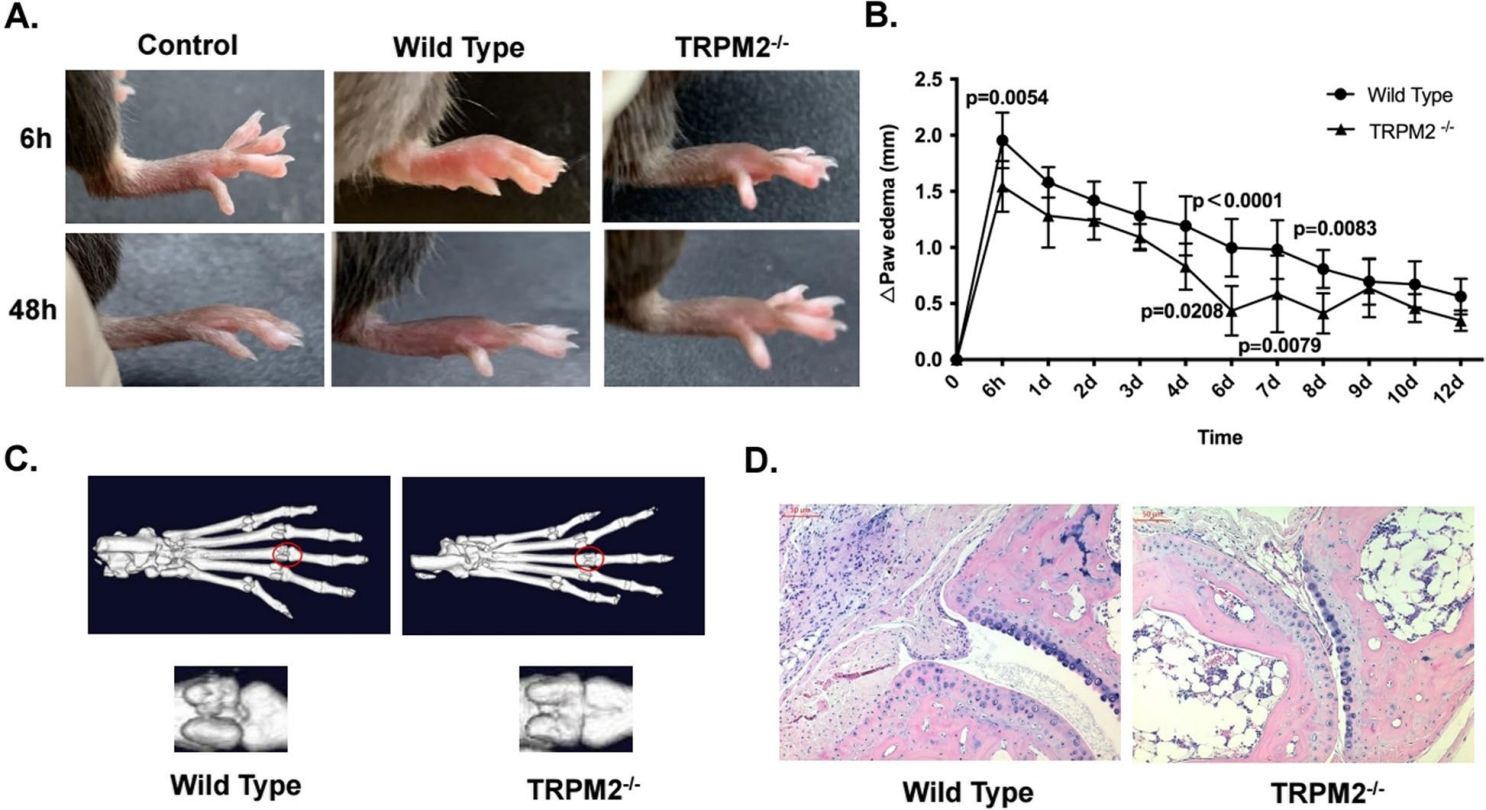
TRPM2 deficiency alleviates paw inflammation and edema induced by MSU crystals. **A** Representative photographs of paw edema (swelling) after injection into WT and TRPM2^−/−^ mice of 0.6 mg MSU crystals in 30 μl PBS. Injection of sterile PBS served as controls. **B** The graph shows the paw edema of WT and TRPM2^−/−^ mice 6 h and 1-12 d after injection of the MSU crystals analyzed by an electronic vernier scale. Mean ± SD is displayed. The *p* values at 6 h, 4d, 6d, 7d and 8d are 0.0054, 0.0208, <0.0001, 0.0079 and 0.0083, respectively. **C** Representative μCT images of WT and TRPM2^−/−^ mice at d 12. **D** Representative H&E staining of the paws of WT and TRPM2^−/−^ mice at d 12. Scale bars, 50 μm

To further analyze the inflammatory response to MSU crystals in TRPM2^−/−^ mice, we injected 0.6 mg MSU crystals subcutaneously into the paws of WT and TRPM2^−/−^ mice. Then we monitored the paw edema (swelling) over time. Injection of MSU crystals led to rapid but self-limited paw inflammation characterized by redness and swelling. When compared to WT mice, TRPM2^−/−^ mice displayed less swelling and a faster resolution of inflammation (Fig. 4A, B). After sacrificing the mice at day 12, we assessed synovial hyperplasia, inflammatory cells infiltration and bone destruction in paw tissue employing μCT and histology. Areas of bone hyperplasia were apparent in WT, but not in TRPM2^−/−^ mice (Fig. 4C). Furthermore, TRPM2^−/−^ mice exhibited less inflammatory cell infiltration and little synovial hyperplasia (Fig. 4D). Together, these results indicate that TRPM2^−/−^ ameliorated inflammation also in the MSU crystals-induced paw edema model.

## Discussion

NET formation can be triggered by a wide range of stimuli in vitro and in vivo during various pathophysiological conditions [22]. In addition to bacterial, fungal, parasitic and viral pathogens, host-derived endogenous inflammatory mediators such as crystals, cytokines, chemokines, immune complexes and autoantibodies and a plethora of further agents are potent inducers of NET formation [23–27]. Previous studies have shown that MSU-crystals induced NET formation is preceded by elevated cytoplasmic calcium levels and, consequently, by the generation of ROS [21, 28]. As important intracellular messenger, high cytosolic Ca^2+^ levels increase the activity of protein kinase C (PKC) which phosphorylates gp91phox. The latter activates NADPH oxidase to generate ROS and thus initiates the downstream signaling that finally results in oxidative burst and NET formation. Furthermore, high cytosolic Ca^2+^ levels activate PAD4, cause histone citrullination and thus promote chromatin depolymerization [29, 30]. However, the mechanisms by which MSU crystals trigger NET formation are still elusive.

Here, we show that TRPM2, a nonselective cation channel, mediated MSU crystals- induced NET formation by regulating calcium influx and ROS production. Deletion of TRPM2 impairs the capacity of neutrophils to form NETs and aggNETs in response to MSU crystals; TRPM2^−/−^ neutrophils displayed reduce cytoplasmic calcium levels and ROS production. This is consistent with the mechanism described for H2O2-mediated NET formation [31]. Deletion of TRPM2 protects against the inflammation caused by MSU crystals.

MSU crystals, one of the prototypic endogenous DAMPs, act as danger signal and elicit robust inflammation [32]. After uptake of MSU crystals, mononuclear phagocytes like monocytes and macrophages engage the caspase-1-activting NALP3 inflammasome [33] to process pro-IL1β and to generate the active form of the proinflammatory cytokine IL-1β, which consequently triggers a feverish inflammation and recruits further neutrophils [9]. The latter vigorously ingest MSU crystals and release large amounts of pro-inflammatory cytokines and chemokines, including TNF-α and IL-6. This precipitates the acute inflammatory reactions and initiates the activation of the adaptive immune response [34]. MSU crystals-induced NET formation also contributes to the resolution of the inflammatory process. Indeed aggNETs promoted the resolution of the MSU crystal-induced inflammation by promoting the proteolytic degradation of inflammatory cytokines and chemokines.

In Ncf1** mice that show an impaired NOX2-dependent generation of ROS and a reduced formation of aggNETs, the neutrophil-driven inflammation in response to MSU crystals exacerbated and became chronic [10]. Here, in MSU crystals-induced murine air pouch inflammation, TRPM2^−/−^ mice showed reduced infiltration of inflammatory cells as well as reduced concentrations of pro-inflammatory mediators in their air pouches. Similar results were to be observed in the neutrophil-driven, MSU crystal-induced paw edema model. Again, the inflammation was alleviated in TRPM2^−/−^ mice.

Previous studies showed that although deficient NET formation delayed the inflammatory responses it also abrogated the resolution of inflammation. In contrast, TRPM2^−/−^ mice that preserved some ROS production and NET formation showed an overall alleviation of the MSU crystal-induced inflammation with intact resolution. Comparing our results to those of older studies, it must be pointed out that Ncf1^**^ mice which carry a single mutation of the neutrophil cytosolic factor 1 (encoded by Ncf1), completely lose the ability for NOX2- dependent ROS generation. In this study, TRPM2^−/−^ mice that have been generated by the deletion of the third and fourth transmembrane domains of the channel protein, produce some ROS and still form (reduced numbers of) NETs. Importantly, these NETs differ morphologically from those of wild type mice.

TRPM2 is highly expressed in many kinds of immune cells [35]. Thus, the deletion of TRPM2 in other immune cells, e.g. macrophages may indirectly affect neutrophils as TRPM2 is involved in the cytokines production of mononuclear phagocytes [36]. Published studies on the role of TRPM2 in neutrophils are quite conflicting. This is an example of the subtle balance the innate immune system needs to cope with the plethora of potential invaders that must reliably be identified and ignored or fended off. Depending on the types of disease models under investigation, TRPM2 deletion consequently leads to an increased [37] or decreased [11, 38–40] production of pro-inflammatory cytokines. However, most studies consider TRPM2 a potential therapeutic target for antagonizing oxidative stress-related pathological conditions such as diabetes, inflammation, neurodegeneration, cardiovascular disease, and stroke [16, 41]. Accordingly, we propose that TRPM2^−/−^ mice alleviate neutrophil-driven inflammation by a combination of altered NETs function and reduced release of inflammatory mediators.

## Conclusion

Overall, our results indicated that mice with targeted deletion of the TRPM2 gene show ameliorated MSU crystal-induced inflammatory responses. Mechanistically, TRPM2 modulated MSU-induced NET formation, resulting in reduced infiltration of inflammatory cells into affected tissues and reduced production of inflammatory mediators. Our results suggest that pharmacological targeting of TRPM2 represents a potential new approach for the treatment of neutrophilic inflammations.

## Data Availability

The data supporting the conclusions of this article are included within the article and its additional file.
